# Late-Onset Neonatal Cyanosis Secondary to Methemoglobinemia in a Preterm, Small-for-Gestational-Age Infant: A Case Report

**DOI:** 10.7759/cureus.116196

**Published:** 2026-09-13

**Authors:** Pooja Patel, Dipika Bhil

**Affiliations:** 1 Department of Pediatrics, Dhiraj Hospital, Smt. B.K. Shah Medical Institute and Research Centre (SBKSMIRC), Sumandeep Vidyapeeth Deemed to be University, Vadodara, IND

**Keywords:** methemoglobinemia, methylene blue, neonatal cyanosis, preterm infant, vitamin c

## Abstract

Methemoglobinemia is a rare cause of neonatal cyanosis stemming from hemoglobin iron oxidation, creating susceptibility due to immature enzymatic systems in newborns. This case study details a 32-week preterm infant presenting with unexplained desaturations (oxygen saturation (SpO2): 82-90%) on day of life 13, which were resolved with intravenous methylene blue and oral vitamin C after co-oximetry confirmed 18% methemoglobin. Prompt diagnosis and low-dose targeted therapy are essential for managing this condition in fragile, preterm neonates with persistent central cyanosis.

## Introduction

Methemoglobinemia is a rare hematological disorder characterized by the oxidation of ferrous iron (Fe2+) within hemoglobin to the ferric state (Fe3+), forming methemoglobin. This structural shift renders methemoglobin incapable of binding oxygen while increasing the oxygen affinity of the remaining normal ferrous globins. The resulting leftward shift of the oxygen-dissociation curve causes functional tissue hypoxia despite a normal total hemoglobin concentration. The condition is broadly classified into congenital forms, such as autosomal recessive cytochrome b5 reductase 3 (CYB5R3) mutations or autosomal dominant hemoglobin M disease, and acquired forms precipitated by oxidizing exposures [1,2]. However, in clinical practice, particularly within neonatal populations, establishing a definitive etiology can be challenging, and cases may present with overlapping risk factors or remain cryptogenic.

Neonates and infants under four months of age possess distinct physiological vulnerabilities to oxidative stress. These include a transient physiological deficiency of nicotinamide adenine dinucleotide (NADH) cytochrome b5 reductase (CYB5R) (at roughly 50% of adult levels), an increased susceptibility of fetal hemoglobin to oxidation, and an elevated gastric pH that facilitates the proliferation of nitrate-reducing bacteria. Clinical symptoms scale with methemoglobin percentages and can be severely compounded by neonatal comorbidities such as sepsis, metabolic acidosis, or severe dehydration. Consequently, a high index of clinical suspicion is essential when a neonate presents with unexplained, generalized cyanosis or hypoxemia that fails to respond to conventional supplemental oxygen therapy [3,4].

Herein, we present the case of a preterm (32 weeks), small-for-gestational-age female neonate who developed late-onset, unexplained desaturations on day of life (DOL) 13. Refractory to supplemental oxygen, she was diagnosed with an elevated methemoglobin level of 18% of unknown definitive etiology and was successfully treated with low-dose intravenous methylene blue and oral vitamin C.

## Case presentation

A preterm (32 weeks), small-for-gestational-age female neonate weighing 1.15 kg was born via normal vaginal delivery at a private facility and transferred to our tertiary neonatal intensive care unit (NICU) on her first DOL due to respiratory distress. Upon admission, physical examination revealed central cyanosis with a preductal oxygen saturation (SpO2) of 82% on room air and a Silverman-Andersen score [5] of 7/10. She was immediately stabilized on continuous positive airway pressure (CPAP) with a positive end-expiratory pressure (PEEP) of 6 mmHg and a fraction of inspired oxygen (FiO2) of 40%. Empirical first-line intravenous antibiotics and maintenance fluids were initiated. Baseline laboratory evaluations revealed neonatal polycythemia, characterized by a hemoglobin of 22.1 g/dL and a packed cell volume (PCV) of 66.2%; sepsis screen was negative. Intravenous fluid administration was increased to 120% of baseline maintenance requirements (100 mL/kg/day) to manage the polycythemia. The patient demonstrated rapid clinical improvement, transitioning to a heated humidified high-flow nasal cannula within eight hours, and was completely weaned to room air by DOL 2. Enteral feeds utilizing expressed breast milk were introduced, tolerated well, and advanced to full volume by DOL 4. Feeds were optimized with standard nutritional supplements, including a multi-component human milk fortifier (HMF) to meet the recommended daily allowances for preterm macronutrients, calcium, and phosphorus, alongside vitamin D supplementation. Concurrently, Kangaroo Mother Care (KMC) was routinely practiced, and her SpO2 remained stable between 90% and 95% on room air.

**Figure 1:**
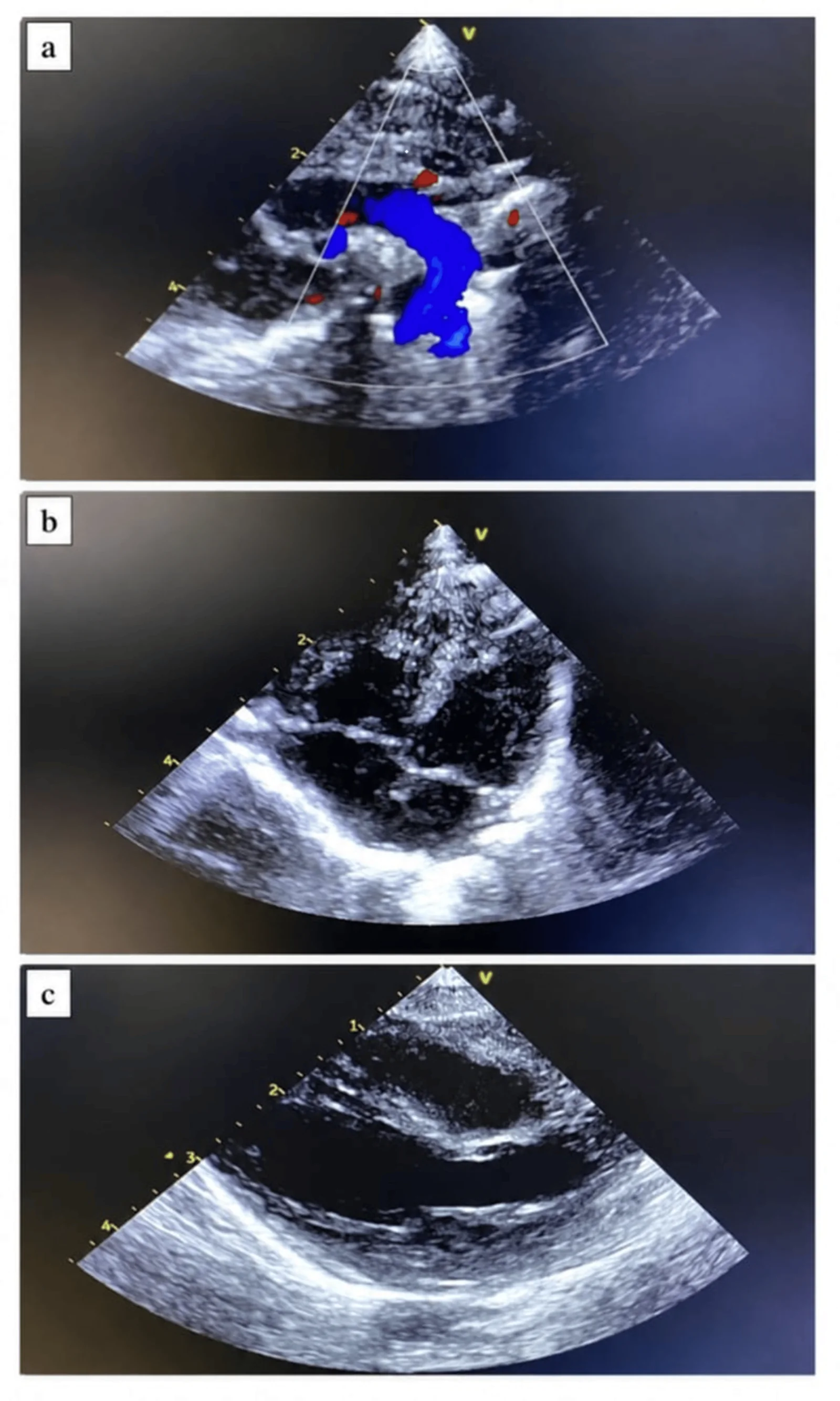
2D echocardiography report (a) Apical four-chamber view with color Doppler showing laminar flow across the atrial septum and ventricular inflow; no evidence of septal defect or valvular regurgitation. (b) Apical four-chamber view in grayscale showing normal cardiac chamber size and configuration with intact interatrial and interventricular septum. (c) Parasternal long-axis view showing normal left ventricular size and systolic function; aortic and mitral valves appear normal with no evidence of regurgitation. Overall impression: normal echocardiographic study.

On DOL 13, the neonate developed sudden, recurrent, and unexplained episodes of desaturation, with SpO2 plateauing between 82% and 90% that proved entirely refractory to supplemental oxygen therapy. Physical examination revealed generalized central cyanosis but was otherwise unremarkable; there were no audible cardiac murmurs, signs of increased work of breathing, or altered perfusion. Simultaneous pre- and post-ductal pulse oximetry demonstrated no saturation gradient. To systematically rule out more common cardiopulmonary etiologies of neonatal cyanosis, an urgent, bedside real-time 2D transthoracic echocardiogram (Figure 1) was performed, which confirmed normal structural cardiac anatomy and function, effectively ruling out cyanotic congenital heart disease and persistent pulmonary hypertension of the newborn (PPHN).

Given the refractory central cyanosis, the absence of a pre-/post-ductal gradient, and the exclusion of primary cardiopulmonary disorders, methemoglobinemia was highly suspected. Blood gas co-oximetry performed on DOL 15 definitively confirmed an elevated methemoglobin level of 18% (Table 1) against a total hemoglobin level of 18.5 g/dL, yielding an absolute methemoglobin concentration of 3.33 g/dL. The carboxyhemoglobin level was normal at 0.2%. Formal arterial blood gas (ABG) analysis was not performed, as the patient remained clinically stable and was managed strictly via close, non-invasive clinical evaluation. A strict medication audit confirmed no exposure to known oxidizing triggers.

**Table 1:** Co-oximetry evaluation on day of life (DOL) 15

Test parameter	Result	Reference range	Analytical method
Methemoglobin (MetHb)	18%	<0.8% of total hemoglobin (Hb)	Blood gas co-oximetry
Carboxyhemoglobin (COHb)	0.2%	0.5-1.5%	Blood gas co-oximetry
Total hemoglobin (Hb)	18.5 g/dL	13.5-18.5 g/dL	Blood gas co-oximetry

Targeted management was initiated immediately on DOL 15 with a single low dose of intravenous 1% methylene blue administered at 0.2 mg/kg/dose, supplemented with oral vitamin C syrup (200 mg/day) for seven days. The neonate exhibited a prompt clinical response; central cyanosis completely resolved, and preductal SpO2 stabilized at 95% on room air.

While the clinical diagnosis of methemoglobinemia was securely established via co-oximetry and confirmed by this rapid therapeutic response, definitive etiological testing was not performed. Due to institutional resource limitations and the swift, complete resolution of the episode without recurrence, specialized confirmatory testing, including CYB5R3 enzyme activity assays, genetic sequencing, and hemoglobin-variant analysis (for hemoglobin M disease), was omitted. This clearly distinguishes her definitive clinical presentation from a confirmed genetic or molecular etiology. Following stabilization, the infant resumed steady weight gain and was discharged on DOL 30 at a weight of 1.5 kg with maintenance oral vitamin C drops (50 mg/day). Prior to discharge, routine neonatal screening protocols were completed: retinopathy of prematurity (ROP) screening demonstrated stage 2 ROP in zone 2 in both eyes with no plus or pre-plus disease, and a bedside otoacoustic emissions (OAE) hearing assessment confirmed normal neurosensory status.

## Discussion

Congenital methemoglobinemia is a rare and potentially life-threatening cause of neonatal cyanosis [6]. The condition arises when the heme moiety of hemoglobin undergoes oxidation, shifting iron from its physiological ferrous state (Fe2+) to an abnormal ferric state (Fe3+) [7,8]. In this oxidized form, hemoglobin is rendered incapable of binding, transporting, and delivering oxygen to peripheral tissues. Under normal erythrocyte metabolism, methemoglobin is continuously generated but rapidly reduced via the enzymatic activity of NADH CYB5R. This homeostatic mechanism strictly maintains physiological methemoglobin levels below 1% of total hemoglobin. However, when the balance between these oxidation and reduction pathways is disrupted, methemoglobin accumulates past the pathological threshold of 3%, manifesting clinically as methemoglobinemia [8].

When a neonate presents with persistent cyanosis in the absence of an identifiable cardiac or pulmonary etiology, clinicians must maintain a high index of suspicion for methemoglobinemia. Confirming this diagnosis in the neonatal period begins with measuring methemoglobin levels to determine if and when therapeutic intervention should be initiated. However, obtaining an accurate quantification in this population poses distinct diagnostic hurdles. Standard pulse oximetry fails to differentiate between methemoglobin, oxyhemoglobin, and deoxyhemoglobin. In contrast, multi-wavelength co-oximetry can isolate methemoglobin by targeting its specific light absorption profile at 630 nm. While the Evelyn-Malloy assay remains the most accurate method for definitive detection, its limited availability typically precludes a timely clinical diagnosis [1,4]. Furthermore, accurate measurement is frequently compromised by other substances that absorb light near the 630 nm wavelength; consequently, the elevated concentrations of fetal hemoglobin, bilirubin, and lipids typical in neonates can obscure results [9,10].

A definitive etiological distinction between congenital and acquired methemoglobinemia remains a profound diagnostic challenge when advanced molecular resources are unavailable. In this case, no genetic sequencing, hemoglobin-variant testing, or erythrocyte enzyme assays (such as CYB5R activity) were performed. Consequently, a congenital etiology, such as autosomal recessive CYB5R gene mutations or autosomal dominant hemoglobin M variants, cannot be definitively established. Hemoglobin M variants involve single amino acid substitutions in α-, β-, or γ-globins that stabilize heme iron in the ferric state, making it resistant to enzymatic reduction. Patients with these variants, including those with the γ-globin variant Hb F-M-Fort Ripley, typically maintain elevated methemoglobin levels of 30-40% but often remain asymptomatic due to chronic physiological compensation [11,12].

The infant's clinical phenotype presents a complex diagnostic picture. While neonatal polycythemia (PCV: 66.2%) suggests a compensatory response to a long-standing congenital insufficiency, the initial satisfactory SpO2 on room air followed by recurrent desaturation on DOL 13 strongly points toward an acquired etiology. Acquired methemoglobinemia can be triggered by external oxidants, including maternal or neonatal medications (topical anesthetics, prilocaine, benzocaine, sulfonamides), feeding-related exposures (nitrate-contaminated well water in formula), or environmental toxins [13]. Although history revealed no explicit toxic exposures, the lack of quantitative toxicological screenings means an acquired etiology cannot be completely excluded. Consequently, the etiology must remain classified as undetermined, spanning both congenital and acquired possibilities.

Therapeutic intervention for methemoglobinemia is typically reserved for symptomatic patients with methemoglobin levels exceeding 10%. In cases driven by a CYB5R deficiency or clear acquired triggers, intravenous methylene blue serves as the primary treatment line [6]; it is conventionally administered at a dose of 0.5-2 mg/kg over five minutes and can be repeated after one hour if the initial reduction is insufficient. Alternatively, when methylene blue is unavailable or contraindicated, ascorbic acid may be utilized as a secondary therapeutic agent [1,10].

In this unique case involving a vulnerable 32-week preterm neonate, a cautious, off-label "low dose" of 0.2 mg/kg of methylene blue was intentionally selected. This reduced dosing strategy aimed to mitigate the well-documented risks of paradoxical oxidizing toxicity, worsening cyanosis, and severe hemolytic anemia associated with standard or higher doses (>4 mg/kg) in low-birth-weight, preterm infants [14]. To augment this conservative approach, oral vitamin C (200 mg/day for seven days) was introduced alongside the initial methylene blue dose to provide sustained, safe antioxidant coverage through an alternative, non-enzymatic reduction pathway.

Although the infant recovered, the simultaneous administration of low-dose methylene blue and ascorbic acid introduces a therapeutic confounder, making it impossible to isolate each drug's contribution or rule out spontaneous resolution. Furthermore, the lack of serial quantitative methemoglobin measurements pre- and post-treatment prevents objective tracking of biochemical clearance, leaving clinical improvement as the sole surrogate for response. Ultimately, while this case provides valuable insight into alternative, low-dose strategies for vulnerable preterm infants, conclusions must remain tempered due to data constraints. In resource-limited settings, clinicians must balance aggressive protocols against patient vulnerability, initiating targeted management based on clinical presentation while acknowledging the diagnostic ambiguities inherent without confirmatory molecular testing.

## Conclusions

Neonatal methemoglobinemia is a vital differential diagnosis for persistent cyanosis when cardiac and pulmonary etiologies are excluded. Because standard pulse oximetry is insufficient for detection, prompt multi-wavelength co-oximetry is essential for accurate quantification. In this case, favorable clinical improvement and resolution of cyanosis were observed following the concurrent administration of low-dose intravenous methylene blue and oral ascorbic acid. However, given the lack of serial biochemical measurements and confirmatory molecular testing, the precise etiology remains undetermined, and the independent efficacy or safety of this specific combination strategy cannot be definitively established from a single observation. Ultimately, maintaining a high index of clinical suspicion and utilizing targeted screening are paramount to achieving positive outcomes in vulnerable neonates.
